# Interplay between cell cycle and autophagy induced by boswellic acid analog

**DOI:** 10.1038/srep33146

**Published:** 2016-09-29

**Authors:** Anup S. Pathania, Santosh K. Guru, Suresh Kumar, Ashok Kumar, Masroor Ahmad, Shashi Bhushan, Parduman R. Sharma, Priya Mahajan, Bhahwal A. Shah, Simmi Sharma, Amit Nargotra, Ram Vishwakarma, Hasan Korkaya, Fayaz Malik

**Affiliations:** 1 Departments of Cancer Pharmacology, Natural Products Microbes; Indian Institute of Integrative Medicine, Canal road Jammu, Jammu and Kashmir, 180001, India; 2 Academy of Scientific and Innovative Research (AcSIR), New Delhi, 110001, India; 3 Discovery Informatics and Natural Products Microbes; Indian Institute of Integrative Medicine, Canal road Jammu, Jammu and Kashmir, 180001, India; 4 Natural Products Microbes; Indian Institute of Integrative Medicine, Canal road Jammu, Jammu and Kashmir, 180001, India; 5 Department of Biochemistry and Molecular Biology, Georgia Regents University Cancer Centre, 1410 Laney Walker Boulevard CN2136, Augusta, GA, 30912, USA

## Abstract

In this study, we investigated the role of autophagy induced by boswellic acid analog BA145 on cell cycle progression in pancreatic cancer cells. BA145 induced robust autophagy in pancreatic cancer cell line PANC-1 and exhibited cell proliferation inhibition by inducing cells to undergo G2/M arrest. Inhibition of G2/M progression was associated with decreased expression of cyclin A, cyclin B, cyclin E, cdc2, cdc25c and CDK-1. Pre-treatment of cells with autophagy inhibitors or silencing the expression of key autophagy genes abrogated BA145 induced G2/M arrest and downregulation of cell cycle regulatory proteins. It was further observed that BA145 induced autophagy by targeting mTOR kinase (IC_50_ 1 μM), leading to reduced expression of p-mTOR, p-p70S6K (T389), p-4EBP (T37/46) and p-S6 (S240/244). Notably, inhibition of mTOR signalling by BA145 was followed by attendant activation of AKT and its membrane translocation. Inhibition of Akt through pharmacological inhibitors or siRNAs enhanced BA145 mediated autophagy, G2/M arrest and reduced expression of G2/M regulators. Further studies revealed that BA145 arbitrated inhibition of mTOR led to the activation of Akt through IGFR/PI3k/Akt feedback loop. Intervention in IGFR/PI3k/Akt loop further depreciated Akt phosphorylation and its membrane translocation that culminates in augmented autophagy with concomitant G2/M arrest and cell death.

Autophagy is a self-degradative lysosomal mediated process used by cells to remove misfolded or aggregated proteins, damaged organelles or intracellular pathogens. Autophagy plays an important role in maintaining cellular homeostasis during stress and has been involved in various cellular processes like DNA repair^1^, angiogenesis^2^, metastasis^3^, Reactive oxygen species (ROS)^4^, inflammation^5^ and cell cycle progression^6^. Dysregulation in any of these process can lead to various types of diseases including cancer^7^. Autophagy is persistently activated in rapidly growing tumors allowing their survival during high metabolic demand and nutrient starvation. However, excessive autophagic flux may also leads to cell death, known as autophagic cell death or type II programmed cell death^8^. Due to its bifunctional roles, modulating autophagy in cancer cells could have better therapeutic benefits.

Studies have demonstrated the direct association between cancer and cell cycle progression due to the gain of function (oncogenes) or loss of function (tumor suppressor genes) of cell cycle regulatory genes^9^. The main cell cycle regulatory proteins are cyclin dependent kinases or CDKs that are positively regulated by cyclins and negatively by CDK inhibitors. Chronological activation of different CDKs and their respective cyclins progress cells through G1, S, G2 or M phases of cell cycle. Genetic alterations in CDKs and their regulatory cyclins or CDK inhibitors leads to hyper activation of CDKs that results in abnormal cell proliferation and cancer^9^. Many anticancer therapies are aimed to target CDKs or their regulators to inhibit tumor growth^10^.

In cancers, the crosstalk between cell cycle progression and autophagy is not clear and needs to be explored further. In accordance to the earlier reports, cells undergoing mitosis are more resistant to autophagy stimuli including starvation and mTOR inhibition^11^. Reduction in the process of autophagy is associated with the decreased activity of type III PI3Kinase subunit, VPS34, an important regulator of autophagy. In mitotic cells VPS34 gets phosphorylated by CDK1 or CDK5 at its threonine 159 residue, which inhibits its interaction with Beclin 1 thus blocking the formation of active Beclin-VPS34-VPS15 complex^12^. Furthermore, inhibition of CDK2 or CDK4 in breast carcinoma cell lines or overexpression of p27 in mouse embryonic fibroblasts induces autophagy^13^. Tasdemir and co-workers have shown that autophagy induced by variety of stimuli (nutrient starvation or chemical inducers like rapamycin, lithium, tunicamycin etc.) has maximal effects in G1 and S phases of cell cycle as compared to G2, determined by simultaneous monitoring of cell cycle and autophagy markers during autophagy induction^14^. Similarly, it has been observed that autophagy also regulates cell cycle progression and growth of cells^15^. Autophagy promotes normal cell division in the budding yeast *Saccharomyces cerevisiae* in nutrient starvation. Autophagy dependent supply of amino acids during starved conditions promotes normal cell cycle progression and maintains genomic stability. Defects in autophagy genes cause abnormal mitosis and increased frequency of aneuploidy in budding yeast under starvation^6^. Additionally, autophagy acts as an effector mechanism of senescence in cells and many autophagy genes are up regulated during this process. Genetic silencing of Atg5 and Atg7 inhibits autophagy and delays senescence^16^.

In the course of study, we have explored the role of autophagy induced by a potent natural product boswellic acid analog (BA145)^17^ on cell cycle progression in pancreatic cancer cell line PANC-1. Boswellic acids have been reported for various biological properties including anti-inflammatory^18^, anti-angiogenic^19^, anti-arthritic^20^ and anti-cancer^21^. Our group has recently shown the anti-cancer, apoptotic and autophagy inducing potential of BA145 in various cancer cell lines^22^,^23^. In this study, we found that BA145 induced robust autophagy in pancreatic cancer cell line PANC-1 in time and dose dependent manner. BA145 triggered autophagy caused G2/M arrest in PANC-1 cells and inhibited cell growth. Induction of autophagy was associated with inhibition of mTOR kinase by BA145, which however led to feedback activation of Akt via IGF-1R/PI3K signalling. Feedback activation of Akt subdued BA145 triggered autophagy and its effect on cell cycle arrest and cell death in PANC-1 cells.

## Results

### BA145 inhibits cell proliferation and induces G2/M arrest in pancreatic cancer cell line PANC-1

**Figure 1:**
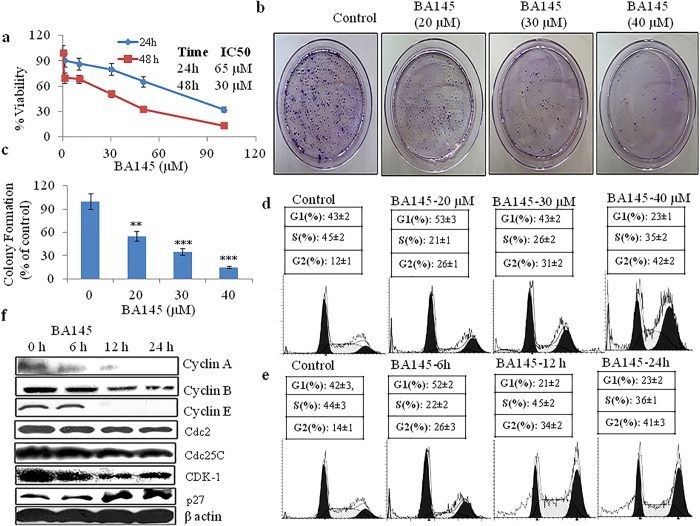
The inhibitory effect of BA145 on cell proliferation, clonogenic survival and G2/M progression in PANC-1 cells. (**a**) MTT cell viability assay of PANC-1 cells treated with different concentrations of BA145 for 24 and 48 h (**b**,**c**) Influence of BA145 on the number of colony forming PANC-1 cells. Cells were treated with BA145 at indicated concentrations for 24 h. Cells were trypsinized and 1000 viable cells were seeded in 60 mm dishes. Cells were allowed to form colonies for 15 days, stained with 1% crystal violet, and counted manually (**d**) Distribution of cell cycle phases in BA145 treated PANC-1 cells. Cells were treated with indicated concentrations of BA145 for 24 h and analyzed for cell populations using flow cytometry (**e**) Effect of BA145 (40 μM) on cell cycle phase distribution of PANC-1 cells at 6, 12 and 24 h (**f**) Effect of BA145 (40 μM) on cell cycle proteins regulating G2/M progression at indicated time intervals. Columns, mean; bars, SD with ***p < 0.001, **p < 0.01 versus control.

To investigate the cell growth inhibitory potential of BA145 in pancreatic cancer, we first examined the effect of BA145 on cell proliferation and clonogenic survival in PANC-1 cells. BA145 treatment inhibited cell growth of PANC-1 cells in dose and time dependent manner. The IC_50_ values of BA145 in PANC-1 cells were 30 μM and 65 μM for 48 and 24 h time periods, respectively (Fig. 1a). Antitumor activity of BA145 was further examined by *in vitro* clonogenic assay. Clonogenicity of PANC-1 cells were reduced in a concentration-dependent manner after exposure to BA145 (Fig. 1b,c). To explore the mechanism of BA145 mediated cell growth inhibition in PANC-1, cell cycle phase distribution was analyzed by using flow cytometry. Our results demonstrated that BA145 treatment arrested cells in G2/M phase, both dose and time dependent manner. Untreated control cells showed 12% population in G2/M phase that was increased to 26%, 32% and 41% at 20, 30 and 40 μM concentrations of BA145 for 24 h respectively (Fig. 1d). Furthermore, time dependent study of BA145 (40 μM) treated PANC-1 cells revealed that 26% and 34% of cell population was in G2/M phase at 6 h and 12 h time periods when compared to 14% in untreated control (Fig. 1e). BA145 treatment exhibited the inhibition of the expression of G2/M regulatory proteins; cyclin A, cyclin B, cyclin E, Cdc2, Cdc25C and CDK-1 in PANC-1, while the expression levels of p27 were increased with the duration of treatment time. (Fig. 1f).

### BA145 induced cell death is independent of apoptosis in PANC-1 cells

**Figure 2:**
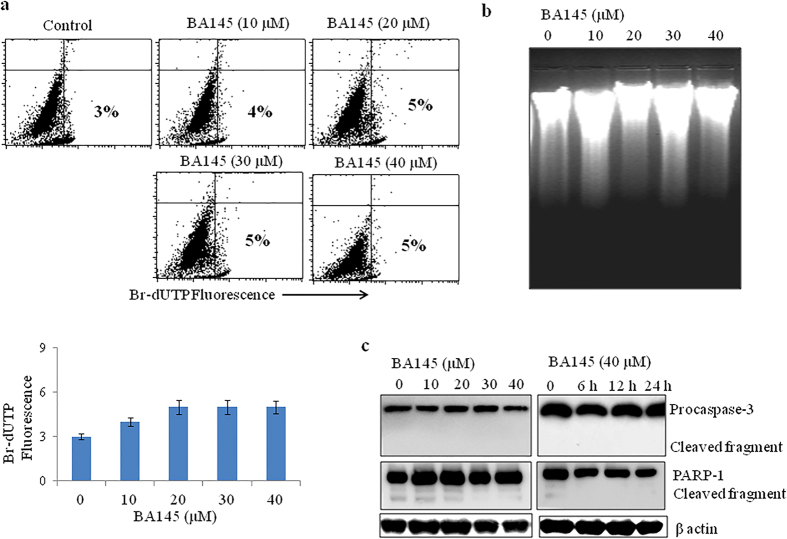
Effect of BA145 on apoptosis induction in PANC-1 cells. BA145 did not induces apoptosis in PANC-1 cells as determined by ApoBrdU assay, DNA fragmentation and western blotting (**a**) Cells were treated with different concentrations of BA145 for 24 h and then stained with ApoBrdU DNA fragmentation kit (APO-DIRECT™ Kit). The apoptotic BrdU positive cells were examined by flow cytometry (**b**) DNA fragmentation assay. Genomic DNA was extracted from BA145 treated cells at indicated doses for 24 h and DNA fragmentation was examined by agarose gel electrophoresis (**c**) Effect of BA145 on the cleavage of procaspase-3 and PARP-1 in PANC-1. Cells were treated with BA145 at indicated doses and time intervals, whole cell lysates were prepared and proteins are resolved on SDS gel for western blot analysis. β-actin was used as a loading control.

Our previously published results have shown that BA145 induced apoptosis in many cancer cells^22^. So, we tried to explore the role of apoptosis in BA145 mediated cytotoxicity in PANC-1 cells. We used APO-BrdU assay to examine the effect of BA145 on DNA damage in PANC-1 cells. APO-BrdU assay showed that BA145 treatment did not induced single or double stranded DNA nicks in PANC-1 cells even at higher (40 μM) concentration (Fig. 2a). These results were further confirmed by DNA fragmentation assay using gel electrophoresis, a typical marker of apoptosis in cells. BA145 treatment of PANC-1 cells at 10, 20, 30 and 40 μM concentrations for 24 h failed to induce DNA fragmentation in PANC-1 cells (Fig. 2b). Further, analysis of apoptotic markers by western blotting displayed no cleavage in procaspse-3 and PARP-1 in PANC-1 cells treated with BA145 (Fig. 2c). These results confirmed that BA145 induced cell death in PANC-1 cells is independent of apoptosis.

### BA145 induces robust autophagy in PANC-1 cells

**Figure 3:**
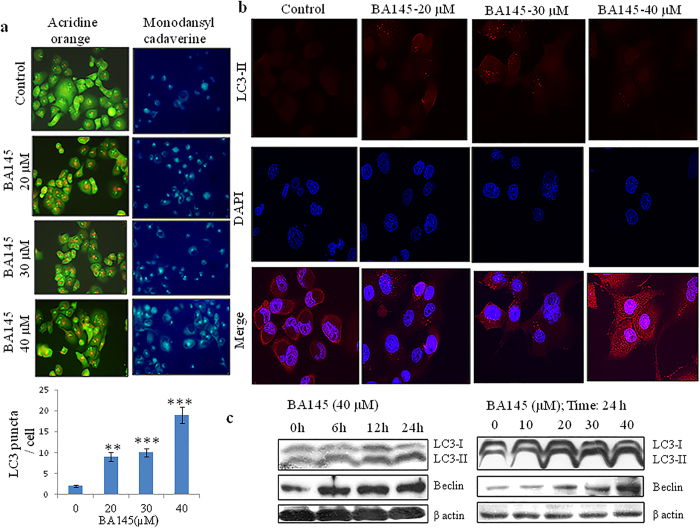
BA145 induces autophagy in PANC-1 cells. BA145 treated cells (20, 30 and 40 μM concentrations; 24 h) were stained with acridine orange (1 μg/ml in serum free media for 10 min) and monodansylcadaverine (50 μM in complete media for 10 min) dyes and fluorescent micrographs were obtained by microscopy. In acridine orange staining, accumulation of acidic vesicular organelle is indicated by the increase in red fluorescence while the untreated control cells are green and in monodansylcadaverine staining, increase in blue florescence indicates acidic vesicle formation (**b**) Detection of autophagy marker LC3-II protein in BA145 treated PANC-1 cells for 24 h by immunofluorescent microscopy (**c**) Western blot analysis of the expression of LC3 and Beclin in BA145 treated PANC-1 cells at indicative concentrations and time intervals. Columns, mean; bars, SD with ***p < 0.001, **p < 0.01versus control.

As BA145 was unable to induce apoptotic cell death in PANC-1 cells; we tried to explore the effect of BA145 on autophagy, an alternative form of cell death also called type II cell death. We performed acridine orange staining to confirm the formation of acidic vesicles in PANC-1 cells by BA145 treatment. Acridine orange has green coloured fluorescence that changes to red after its accumulation inside acidic vesicles. Compared with cells in the basal state, BA145 treatment increased red coloured acridine orange fluorescence in PANC-1 cells in concentration dependent manner. Further, staining with monodansylcadaverine (MDC) dye confirmed the formation of acidic vesicles in PANC-1 cells as increase in MDC fluorescence was observed after BA145 treatment (Fig. 3a). Immunofluorescence staining of widely used autophagy marker, LC3B in PANC-1 cells confirmed the induction of autophagy. As expected, BA145 treatment at 10 μM, 20 μM, 30 μM and 40 μM concentrations increased LC3B fluorescence in cells (Fig. 3b). Western blot analysis of LC3B and Beclin, essential autophagy effectors and central players in autophagosomes formation^24^, revealed a dose and time dependent increase in their expressions with BA145 treatment (Fig. 3c).

### BA145 triggered autophagy arbitrates G2/M arrest in PANC-1 cells

**Figure 4:**
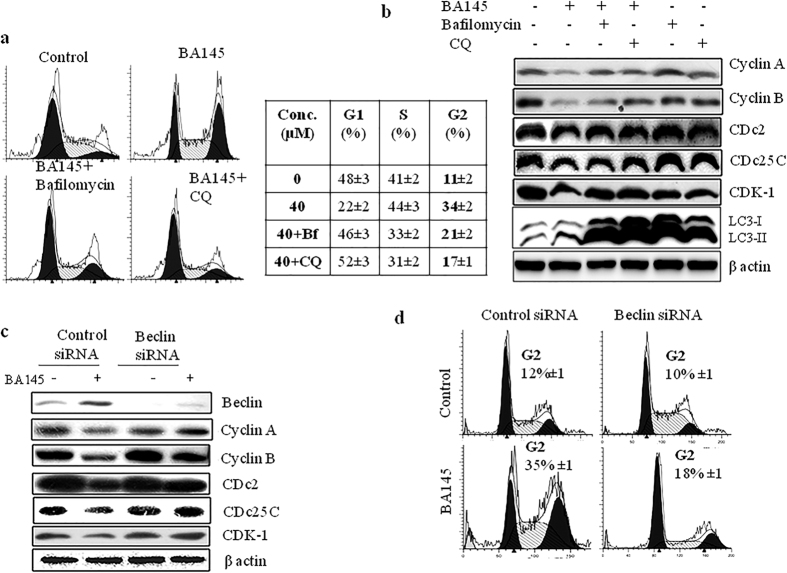
Role of autophagy in BA145 mediated G2/M arrest. (**a**) Pharmacological inhibition of autophagy arbitrated BA145 mediated G2/M arrest in PANC-1 cells. PANC-1 cells were co-treated with BA145 (40 μM) and/or the autophagy inhibitors bafilomycin (10 nM) and CQ (10 μM) for 12 h. Autophagy inhibitors were added 1 h before BA145 treatment. Cells were stained with PI and analyzed for cell cycle phase distribution by flow cytometry (**b**) Effect of autophagy inhibitors on the expression of G2/M regulatory proteins in BA145 treated PANC-1 cells. Cells were treated with BA145 and/or autophagy inhibitors as above described concentrations for 12 h and expression of various proteins were accessed by western blotting (**c**) Influence of beclin inhibition on the expression of G2/M regulatory proteins in BA145 (40 μM) treated PANC-1 cells. After BA145 treatment for 12 h, whole cell protein lysates were prepared for western blotting and the detection of the indicated proteins. β-actin was used as a loading control (**d**) Influence of Beclin inhibition on cell cycle phase distribution in BA145 (40 μM) treated PANC-1 cells for 12 h.

The role of BA145 induced autophagy in cell cycle phase distribution was further explored in PANC-1 cells. We used two well defined autophagy inhibitors bafilomycin and chloroquinone (CQ) to inhibit autophagy in cells. A 12h treatment of BA145 in PANC-1 cells 12 h showed 34% cell population in G2/M phase however, co-treatment with bafilomycin and CQ reduced this population to 21% and 17%, respectively (Fig. 4a). Pharmacological inhibitors of autophagy reversed the expression of of G2/M associated proteins, cyclin A, cyclin B, CDc2, CDc25 and CDK1 (Fig. 4b). Pre-treatment of autophagy inhibitors in BA145 exposed PANC-1 cells, increased LC3B accumulation indicating the induction of autophagic flux (Fig. 4b). Next, we silenced key autophagy gene Beclin in PANC-1 cells using siRNA to evaluate the expression of G2/M regulatory proteins in BA145 treated PANC-1 cells. Silencing of Beclin by siRNA rescued the BA145 mediated inhibition of G2/M regulatory proteins (Fig. 4c). Additionally, Beclin silenced PANC-1 cells showed 18% population in G2/M phase compared to 35% in wild type after BA145 treatments (Fig. 4d).

### BA145 inhibits mTOR signaling in PANC-1 cells

**Figure 5:**
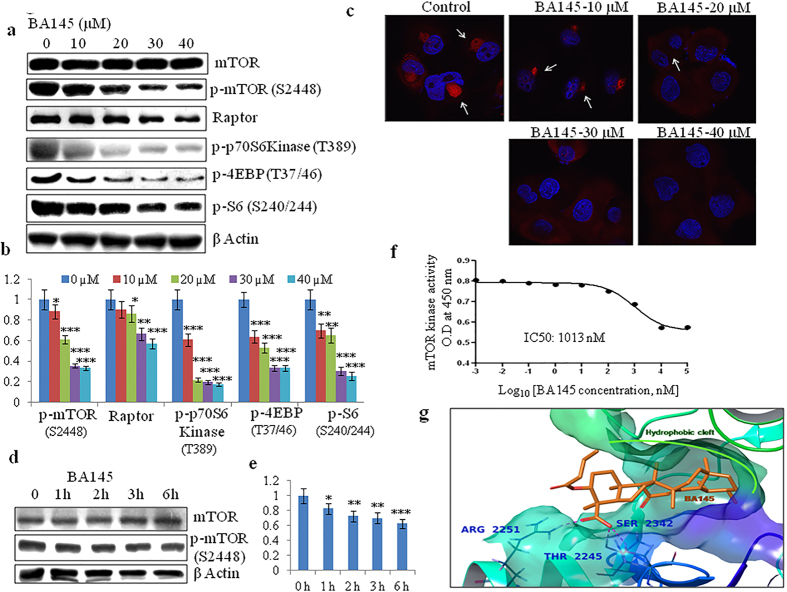
BA145 inhibits mTOR pathway in PANC-1 cells. (**a**) PANC-1 cells were treated with BA145 at indicated concentrations for 12 h. Whole cell protein lysates were prepared and the expression levels of mTOR and its downstream substrates were analyzed by western blotting (**b**) Densitometric analysis of p-mTOR (S2448), Raptor, p-p70S6Kinase (T389), p-4EBP (T37/46) and p-S6(S240/244) protein expression in BA145 treated PANC-1 cells (**c**) Detection of p-mTOR (serine 2448) by immunofluorescent microscopy in BA145 (12 h) treated PANC-1 cells at different concentrations. BA145 treatment inhibits p-mTOR expression (red arrowheads) in PANC-1 cells in dose dependent manner (**d**) PANC-1 cells were exposed to BA145 (40 μM) at 1 h, 2 h, 3 h, 4 h and 6 h time intervals and the expression of mTOR and p-mTOR proteins were examined by western blotting. β-actin was used as a loading control (**e**) Densitometric analysis of p-mTOR expression (**f**) BA145 inhibits mTOR kinase in cell free mTOR kinase enzyme assay (**g**) 3D Interaction diagram of BA145 (in orange) with mTOR. The fused rings of the ligand are enclosed by the hydrophobic cleft formed within the binding pocket and the residues forming H-bond with the carboxylic group of the ligand are also shown in bold. The glide score of the complex was found to be −10.49 Kcal/mol. Columns, mean; bars, SD with ***p < 0.001, **p < 0.01, *p < 0.05 versus control.

mTOR is the central regulator of autophagy under normal conditions^25^ and inhibition of mTOR signaling induces autophagy in many cells^26^. We had previously shown that BA145 inhibited PI3Kinase-Akt-mTOR pathway in leukemic cell line HL-60^22^. In PANC-1 cells, BA145 treatment inhibited activation of mTOR and its downstream targets p70S6kinase and 4EBP-1 in a dose dependent manner. The phosphorylation of S6, a downstream target of p-70S6Kinase was also decreased in cells after BA145 treatment (Fig. 5a,b). Inhibition of mTOR phosphorylation by BA145 was further confirmed by confocal microscopic studies, where red fluorescence of p-mTOR specific antibody (p-mTOR, S2448) was decreased after BA145 treatment in dose dependent manner (Fig. 5c). Additionally, short term BA145 treatment in PANC-1 showed mTOR inhibition as early as 1 h that showed further decreased with time (Fig. 5d,e). Next, we performed cell free enzyme assay to check the effect of BA145 on mTOR kinase activity using K-LISA^TM^ mTOR assay kit (Calbiochem, #CBA055). BA145 inhibited mTOR kinase activity in dose dependent manner with kinase inhibitory IC_50_ value about 1 μM (Fig. 5f). These results were further confirmed by molecular docking studies. Docking studies showed that the binding pocket of mTOR comprised of a hydrophobic cleft, formed by TYR2225, ILE2237, TRP2239, VAL2240, MET2345, LEU2354, ILE2356, ILE2163 and LEU2185, and a solvent accessible region as shown in Supplementary Figure 1a. The most common docking conformation of BA145 within the binding pocket is the one where the fused rings part of BA145 is oriented towards the hydrophobic cleft, which also includes the hinge region (VAL2240). Whereas, the flexible part of BA145, which is formed by the butanoate and the carboxyl group attached to the ring A, is oriented towards the solvent accessible area. In this orientation of BA145, one oxygen of the carboxylic group is involved in H-bonding with ARG2251 at a distance of 1.64 A° and the other oxygen is involved in H-bonding with THR2245 and SER2342 at a distance of 1.99 A° and 2.12 A° respectively as shown in Fig. 5g.

### mTOR inhibition by BA145 is followed by attendant activation of Akt

**Figure 6:**
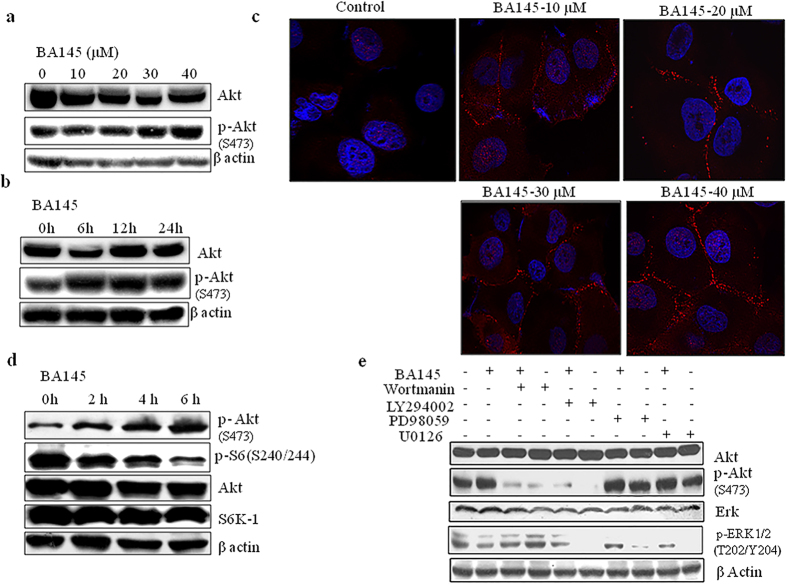
Inhibition of mTOR signaling by BA145 leads to an increase in Akt phoshorylation in PANC-1 cells. (**a**) PANC-1 cells were treated with BA145 at indicated doses for 24 h and then lysed in lysis buffer for western blot analysis phosphorylation and expression of Akt (**b**) Time dependent increase in p-Akt expression (serine 473) in BA145 (40 μM) treated PANC-1 cells (**c**) BA145 induced membrane translocation of Akt (red arrow heads) in PANC-1 cells in dose dependent manner. Cells were treated with BA145 at indicated doses for 24 h and then immunofluorescence staining was performed using fluorescent p-Akt (serine 473) antibody (**d**) Western blot analysis of the Akt, S6 kinase and their phosphorylated forms in BA145 (40 μM) treated PANC-1 cells at indicated time intervals (**e**) Involvement of PI3kinases in BA145 mediated activation of Akt. PANC-1 cells were pre-treated with PI3Kinase inhibitors wortmanin (1 μM) and LY294002 (30 μM) and MEK/Erk pathway inhibitors PD98059 (40 μM) and U0126 (20 μM) for 1 h and then cotreated with BA145 (40 μM) for another 5 h. The cells were subjected to the preparation of whole cell protein lystaes for detection of the indicated proteins using western blotting. PI3kinase inhibitors blocks the phosphorylation of Akt at serine 473 induced by BA145 whereas, Mek/Erk pathway inhibitors have no any effect of Akt activation.

Akt being placed upstream of mTOR is responsible for its activation in many cells types^27^. We evaluated the effect of BA145 on Akt expression in PANC-1 cells. To our surprise, BA145 treatment increased the levels of phosphorylated Akt (serine 473) in cells in time and dose dependent manner (Fig. 6a,b). Further analysis by confocal microscopy revealed that BA145 induced membrane translocation of p-Akt in PANC-1 cells (Fig. 6c). To evaluate the relationship between suppression of mTOR signaling and status of Akt phosphorylation, we exposed PANC-1 cells to BA145 at short time intervals and then examined the activation of Akt and S6 phosphorylation. BA145 treatment inhibited S6 phosphorylation as early as 2 h with subsequent phosphorylation of Akt in PANC-1 cells. These events begun at early time and took place in concurrent manner. However, the expression of total Akt and S6Kinase remain unaffected after BA145 treatment (Fig. 6d). Since Akt is mainly activated by type I PI3Kinases in cells^28^, we tried to find out whether feedback activation of Akt is through PI3K. Two well defined PI3Kinase inhibitors wortmanin and LY294002 were used to inhibit PI3Kinases expression in PANC-1 cells. Our results demonstrated that in absence of wortmanin and LY294002, BA145 induced Akt phosphorylation in PANC-1 cells, whereas the addition of these inhibitors obliterated the activation of Akt. These results suggested that BA145-induced Akt activation requires PI3Kinases. Furthermore, another important regulator of Akt activation in cells is Raf/Mek/Erk signaling and the crosstalk between both pathways is found in many cancers^29^. The inhibition of one pathway leads to the activation of other signaling pathway, thereby decreasing the effectiveness of single target based cancer therapy^30^. To explore the role of Mek/Erk signaling in Akt activation, we used two Mek/Erk pathway inhibitors PD98059 and U0126 along with BA145. Our results demonstrated that Mek/Erk pathway inhibitors do not influence the effect of BA145 mediated Akt phosphorylation in PANC-1 cells, thus ruling out the role of Mek/Erk involvement in the activation of Akt (Fig. 6e).

### Inhibition of Akt phosphorylation increases BA145 mediated autophagy and G2/M arrest

**Figure 7:**
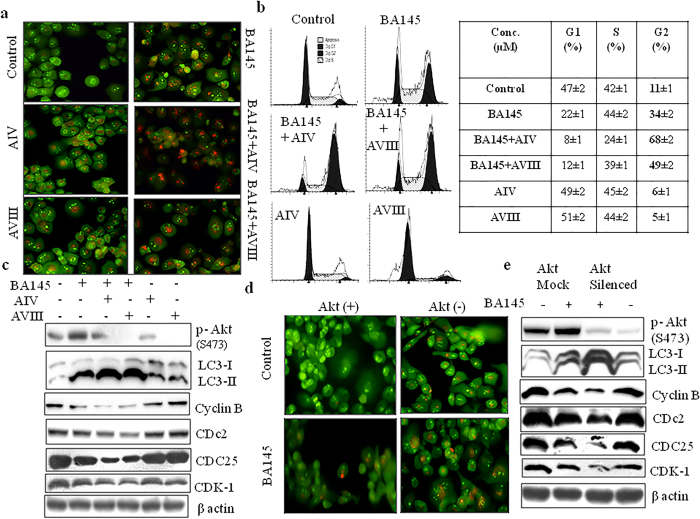
Effect of Akt inhibition on BA145 induced autophagy and G2/M arrest. (**a**) Pharmacological inhibition of Akt augments BA145 induced autophagy. PANC-1 cells were treated with BA145 (40 μM) and/or specific Akt inhibitors Akt inhibitor IV (5 μM) and Akt inhibitor VIII (2 μM) for 12 h time period. Cells were stained with acridine orange (1 μg/ml) for 10 min, and analyzed by fluorescent microscopy. Cotreatment of BA145 with Akt inhibitors enhanced the accumulation of red coloured acidic vesicles in PANC-1 cells (**b**) Effect of Akt inhibitors on cell cycle progression in BA145 treated PANC-1 cells. Cells were treated with BA145 and/or Akt inhibitors at above described concentrations for 12 h time period. Cells were stained with PI and cell cycle populations were analyzed by flow cytometry. Pharmacological inhibition of Akt enhanced cell population in G2/M phase in BA145 treated PANC-1 cells (**c**) Akt inhibitors augmented the inhibitory effect of BA145 on cell cycle regulatory proteins in PANC-1 cells. After BA145 treatment with/or Akt inhibitors, cells were lysed with lysis buffer and protein lysates were prepared for western blotting of indicated proteins. β-actin was used as loading control (**d**) Genetic silencing of Akt through gene specific siRNA enhanced the accumulation acridine orange positive acidic vesicular organelles in BA145 treated PANC-1 cells (**e**) Influence of Akt inhibition by siRNA in the expression of G2/M regulatory proteins in BA145 treated PANC-1 cells. BA145 has more inhibitory effect on the expression of cell cycle regulatory proteins in Akt silenced cells as compared to scrambled siRNA control cells.

To further evaluate the impact of Akt activation on BA145 mediated autophagy and G2/M arrest, we inhibited Akt expression in PANC-1 cells by using two Akt inhibitors, Akt inhibitor IV and Akt inhibitor VIII. Addition of these inhibitors abrogated BA145 induced Akt phosphorylation in PANC-1 cells and enhanced the accumulation of LC3-II levels (Fig. 7c). These results were further confirmed by acridine orange staining showing that the inhibition of Akt signaling increased the formation of acidic vesicles in cells when compared to BA145 alone (Fig. 7a). Since autophagy induced by BA145 causes G2/M arrest, we tried to evaluate the effect of Akt inhibition on G2/M progression in PANC-1 cells. Pharmacological inhibition of Akt enhanced the inhibitory of BA145 on G2/M progression in PANC-1 cells. BA145 treated PANC-1 cells for 12 h time period showed 34% population in G2/M phase that increased to 68% and 49% by co-treatment with Akt inhibitors IV and VIII, respectively (Fig. 7b). It was found that addition of these inhibitors augmented the inhibitory effect of BA145 on G2/M regulatory proteins (Fig. 7c). To strengthen our findings, we silenced Akt in PANC-1 cells by using specific siRNA and checked the effect of BA145 on autophagy and G2/M progression. Silencing of Akt increased BA145 mediated autophagy as analyzed by acridine orange staining and the expression of associated proteins (Fig. 7d,e). Akt knockdown further enhanced the inhibitory effect of BA145 on cyclin B, CDc2, CDC25 and CDK-1 in PANC-1 cells (Fig. 7e). These results indicated that activation of Akt after BA145 treatment resists autophagic process and hence G2/M arrest.

### Activation of Akt in BA145 treated cells is IGF-1R dependent

**Figure 8:**
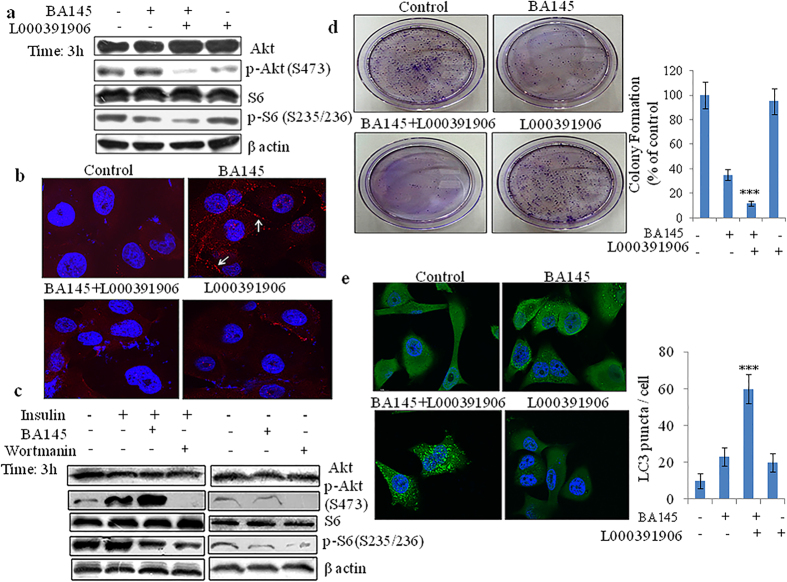
Blockage of IGF/IGF-1R signaling prevents BA145 mediated Akt activation and enhances BA145 induced autophagy. PANC-1 cells were pretreated with specific IGF-1R inhibitor L000391906 (4 μM) for 1 h and then cotreated with BA145 (40 μM) for 3 h. The cells were lysed in lysis buffer and then subjected to western blot analysis of indicated proteins (**b**) Blockage of IGF-1R signaling with L000391906 prevents BA145 mediated membrane translocation of p-Akt. Cells were treated L000391906 (4 μM) for 1 h and then cotreated with BA145 (40 μM) for 3 h. Cells were stained with immunofluorescent p-Akt (Serine 473) antibody and analyzed by fluorescence microscopy (**c**) BA145 induces Akt activation in IGF dependent manner. PANC-1 cells were serum starved overnight and cotreated with either BA145 (40 μM) or wortmanin (1 μM) in presence or absence of IGF activator, insulin (100 ng/ml) for 3 h. Cells were lysed in lysis buffer for western blot analysis of Akt, S6K and their phosphorylated forms. β-actin was used as a loading control (**d**) Effect of L000391906 (4 μM) on the colony forming ability of BA145 (40 μM, 12 h) treated PANC-1 cells (**e**) Inhibition of IGFR-1 signaling enhanced BA145 mediated autophagy in PANC-1 cells. Cells were treated with BA145 (40 μM) and/or L000391906 (4 μM) for 6 h and then after stained with fluorescent antibody and analyzed by immunofluorescent microscopy. Columns, mean; bars, SD with ***p < 0.001 versus BA145.

**Figure 9:**
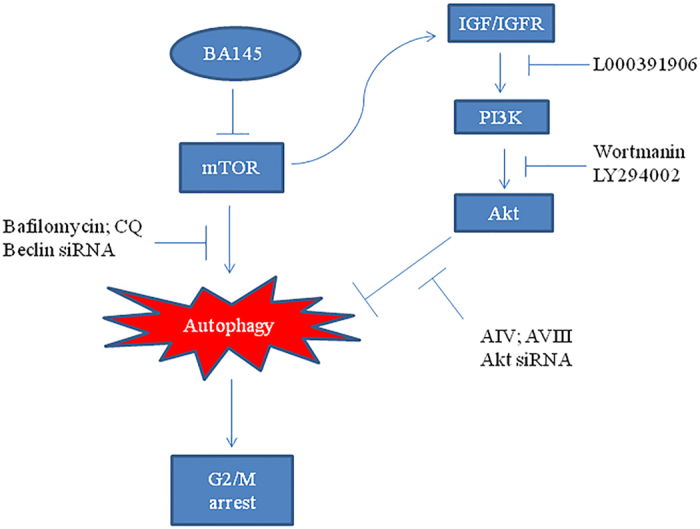
Diagrammatic presentation of the mechanism of cell cycle arrest by BA145 induced autophagy.

Inhibition of mTOR signaling often activates PI3Kinase/Akt pathway as a feedback mechanism in many cancers by up regulating insulin-like growth factor-1 receptor (IGF-IR) signaling^31^. mTOR pathway inhibitors like rapamycin activates such siganling by inducing the expression of insulin receptor substrate-1 (IRS-I), resulting in Akt activation^32^. To determine whether the induction of Akt expression by BA145 is dependent on IGF-IR, we employed small molecule IGF-IR kinase inhibitor, L000391906 having IC50 value of 0.006 μM (Piramal life sciences India). Pre-treatment of L000391906 with BA145 for 3 h time period completely abrogated BA145 induced Akt phosphorylation (Fig. 8a). Confocal microscopic studies revealed that inhibition of IGF-1R prevents BA145 induced Akt membrane translocation in PANC-1 cells (Fig. 8b). To further confirm the role of IGF signaling in BA145 induced feedback Akt activation; we examined the ability of BA145 to stimulate Akt phosphorylation in insulin treated and nontreated PANC-1 cells. In presence of insulin, an activator of IGF-IR signaling, BA145 induced strong Akt phosphorylation in PANC-1 cells whereas the phosphorylation was not significant in absence of insulin. Furthermore, the phosphorylation of S6 was significant in insulin stimulated cells compared to those of unstimulated cells (Fig. 8c). Inhibition of IGF-1R also enhanced the inhibitory effect of BA145 on clonogenic survival of PANC-1 cells (Fig. 8d). Microscopical analysis of LC3 puncta showed that L000391906 co-treatment enhanced the autophagic flux in BA145 treated PANC-1 cells (Fig. 8e), These results thus demonstrated the activation of IGF-IR/AKT feedback loop in PANC-1 cells undergoing autophagy mediated G2/M arrest and cell death during BA145 treatment (Fig. 9).

## Discussion

Pancreatic cancer is one of the most aggressive solid malignancies with around five percent 5 years patient survival rate^32^. Despite decades of efforts, it still remains a devastating disease and resistance often develops against anticancer therapies. Autophagy is one of the mechanisms behind such resistance and inhibiting this process by using pharmacological inhibitors like CQ or hydroxyl CQ or by genetic silencing of key autophagy genes has been used to suppress pancreatic tumor growth *in vitro* and *in vivo*^33^,^34^. Pancreatic tumors have elevated levels of autophagy under basal conditions and it is required for pancreatic ductal adenocarcinoma (PDAC) pathogenesis^34^. Clinic pathological studies of 71 pancreatic cancer patients revealed the direct link of autophagy with poor prognosis of the disease^35^. In contrast, autophagy induced by many anticancer agents acts as a cell death mechanism in pancreatic cancer cells and its inhibition abrogates their cytotoxic properties^36^,^37^,^38^. Autophagy is known for its role in the regulation of cell growth by controlling the cell cycle progress during nutrient deprivation or other cellular stresses^15^. It has also been found that autophagy induces cell cycle arrest in some glioblastoma cell lines, a possible growth inhibitory mechanism in cancer cells^39^. Overexpression of cell cycle regulators p16, p27 and CDK-1 induces autophagy in cells^40^. Further, genetic silencing of key autophagy proteins such as Beclin 1 and Ambra 1 in experimental mice models results in increased cell proliferation^40^. However, there is not much evidence of autophagy regulating cell cycle and less is known about the crosstalk between these two cellular processes. Therefore, in the present study, we explored the role of autophagy induced by natural product analog BA145 in the regulation of cell cycle in pancreatic cancer. We used aggressive and most resistant pancreatic cancer cell line PANC-1 during the course of study^41^,^42^. During preliminary experiments it was found that BA145 induced robust autophagy and inhibited proliferation of PANC-1 cells. BA145 mediated autophagy was found accompanied with mTOR pathway inhibition and arrest of cells in G2/M phase. It was also found that mTOR inhibition by BA145 triggered Akt activation via feedback loop involving IGFR/PI3k signaling. Inhibition of IGFR abrogated BA145 mediated Akt activation with increased autophagic flux and G2/M arrest in PANC-1 cells.

Our results demonstrated that BA145 effectively inhibited cell proliferation and clonogenic survival of PANC-1 cells associated with of cell cycle arrest. BA145 treatment arrested cells at G2/M phase in dose and time dependent manner and effectively decreased the expression of G2/M progression regulatory proteins cyclin A, cyclin B, cyclin E, Cdc25C, and Cdc2 and CDK-1. The expression of tumor suppressor and G2/M regulatory protein^43^, p27 was increased after BA145 treatment. Our previous study showed that BA145 induces robust apoptosis in variety of cancer cell lines^22^. Surprisingly, BA145 did not induce any apoptotic cell death in PANC-1 cells as was observed by several pro-apoptotic parameters like single or double stranded DNA nicks by Apo BrdU assay, DNA fragmentation by DNA ladder pattern and cleavage of apoptotic proteins caspase-3 and PARP-1 by western blotting. BA145 triggered autophagy was confirmed by the accumulation of acidic vesicular organelles in PANC-1 cells as analyzed by acridine orange and MDC staining and increased expression of autophagic proteins beclin and lipidated LC3 (LC3-II). Moreover, LC3-II levels were accumulated when the flux of autophagosomes, from their formation to fusion with lysosomes were inhibited with late autophagy inhibitors CQ and bafilomycin. Next, we investigated the effect of BA145 mediated autophagy on cell cycle progression in PANC-1 cells. Previous reports by Matsui *et al*., 2012^6^ revealed that autophagy induced during starvation arrest cells at G2/M phase in a Swe1-dependent checkpoint mechanism and supply amino acids to cells for their recovery from G2/M delay and hence promotes cell growth^6^. In the course of study, we found that BA145 mediated autophagy regulates G2/M progression in PANC-1 cells. Inhibition of autophagy with pharmacological inhibitors bafilomycin and CQ averted the BA145 induced G2/M arrest as was analysed by flow cytometry. Moreover, BA145 mediated suppression of G2/M regulatory proteins cyclin A, cyclin B, cyclin E, Cdc25C, and Cdc2, CDK-1 were also subdued by suppression of autophagy. siRNA mediated inhibition of the expression of autophagy proteins led to the restoration of G2/M regulatory proteins inhibited by BA145. PI3kinase-Akt-mTOR signaling is the major regulator of autophagy and inhibition of this pathway often triggers autophagy in cancer cells^32^,^44^. Our results demonstrated that BA145 inhibited mTOR phosphorylation in a dose dependent manner as analyzed by western blotting and confocal microscopy. The inhibitory effect of BA145 on mTOR was associated with the down regulation of its downstream targets p-p70S6kinase (T389) and p-4EBP (T37/46). In addition, exposure to BA145 reduced the phosphorylation of ribosomal protein S6, substrate of p70S6kinase in a dose dependent manner. BA145 treatment suppressed mTOR phosphorylation at an early stage of 1 h, which was further decreased with the increased exposure time. Inhibitory effect of BA145 on mTOR kinase activation was further confirmed by cell free mTOR kinase assay. Additionally, molecular docking studies were performed to study the interaction between mTOR and BA145. mTOR being regulated by its upstream activator Akt^27^, we next investigated the effect of BA145 on Akt activation. Since mTOR inhibition is often associated with the feedback activation of Akt in cancer cells and one of the mechanisms of resistance developed against mTOR inhibitors in cancer therapy^32^,^45^. In our study, we found that inhibition of mTOR by BA145 was associated with the subsequent activation of Akt in cells. BA145 treatment induced two fold Akt activation as early as 2 h with concomitant suppression of phosphorylation of p70S6kinase substrate, S6. However, the increase in Akt phosphorylation by BA145 was abrogated by co-treatment with PI3Kinase inhibitors wortmanin and LY294002, suggesting that PI3K activity is required for Akt activation by BA145. Another important mechanism of Akt activation in cells involves Mek/Erk pathway and the crosstalk between these two pathways is found in many cancers^30^,^46^. BA145 treatment suppressed ERK activation in PANC-1 cells. However, co-treatment with Erk inhibitors PD98059 and U0126 did not show any effect on BA145 induced Akt activation thus ruling out the role of this pathway in the activation of Akt. Cellular PI3K/Akt pathway is regulated by upstream transmembrane receptor tyrosine kinases, specifically IGFR. IGFR is activated by insulin and insulin like growth factors (IGFs) thus controls many physiological processes like metabolism, growth and survival in cells^47^,^48^. Our data demonstrated that increase in Akt phosphorylation after mTOR inhibition occurs through IGFs/IGF-1R pathway. Blocking this pathway with highly specific IGFR inhibitor abrogated BA145 induced Akt activation and its membrane translocation. To further confirm our findings, we enhanced IGFR expression in PANC-1 cells by using insulin followed by BA145 treatment for 3 h time period. BA145 induced Akt phosphorylation in PANC-1 cells was stronger in the presence of insulin suggesting the role of IGFs/IGF-1R signaling in the activation of Akt by BA145. Further, it was observed that feedback activation of Akt impaired BA145 triggered autophagy in PANC-1 cells. Inhibition of Akt by using selective pharmacological inhibitors, Akt inhibitor IV and Akt inhibitor VIII or by using Akt specific siRNA augmented the BA145 induced autophagy. Inhibition of IGF1/PI3k/Akt pathway enhanced BA145 mediated autophagy with consequent G2/M arrest in PANC-1 cells.

This study demonstrated that BA145 triggered autophagy inhibited cell proliferation and clonogenic survival of PANC-1 cells by inhibiting mTOR signalling. BA145 induced autophagy caused G2/M cell cycle arrest leading to non-apoptotic death of PANC-1 cells. Furthermore, BA145 arbitrated inhibition of mTOR triggered a negative feedback mechanism resulting in the activation of Akt signaling via IGF-1R/PI3K pathway, thus impeding the autophagy flux and G2/M arrest. Intervention in the IGF-1R/PI3K/Akt pathway ameliorated the BA145 induced autophagic flux and G2/M arrest of PANC-1 cells.

## Material and Methods

DMEM, propidium iodide (PI), acridine orange, monodansyl cadaverine, 3-(4,5-dimethylthiazole-2-yl)-2,5 diphenyltetrazolium bromide (MTT), bafilomycin, chloroquinone (CQ), sodium fluoride, sodium orthovanadate, fetal bovine serum were purchased from Sigma-Aldrich, Missouri, USA. Akt inhibitor IV and VIII, antibodies of caspase-3, cyclin A, cyclin B, cdc2, PARP-1, β-Actin and Akt siRNA were from Santa Cruz Biotechnology, Texas, USA. Antibodies to cdc25, CDK-1, cyclin E were purchased from Piercenet, Illinois, USA. All other antibodies and chemicals were purchased from Cell signaling technology, Massachusetts, USA. Electrophoresis reagents, reagents for protein estimation and protein molecular weight markers were from Bio-Rad Laboratories, California, USA. Polyvinyldifluoride (PVDF) membrane was purchased from Millipore, Massachusetts, USA. K-LISA mTOR activity kit were purchased from Calbiochem, San Diego, USA.

### Cell culture and treatments

Human pancreatic cancer cell line PANC-1 was purchased from ECACC, England. Cells were grown in DMEM growth medium containing 10% FCS, 100 mg/L kanamycin using CO_2_ incubator (Thermocon Electron Corporation, Houston, TX) at 37 °C with 95% humidity. BA145, Bafilomycin, Akti IV, Akti VIII and IFGR inhibitor used in the experiments were dissolved in DMSO (final concentration used in the media is <0.2%) while CQ were solubilised in MQ water.

### Cell proliferation Assay

Cells were seeded in 96 well plates upto 70–75% confluency and were treated with different concentrations of BA145 for 24 and 48 h. MTT dye was added 3 h prior to experiment termination. Cell proliferation assay was performed by using MTT assay (250 μg/ml) as described earlier^22^.

### DNA fragmentation assay

DNA fragmentation assay was used to detect apoptotic DNA damage induced by BA145 in cancer cells. Cells (75% confluency) were treated with BA145 at different concentrations for 24 h. Cells were washed with PBS and lysed in 250 μl of lysis buffer (100 mM NaCl, 5 mM EDTA, 10 mM Tris–HCl; pH 8.0, 5% Triton X-100 and 0.25% SDS) for 10 min followed by RNAse (200 μg/ml) incubation for 2 h at 37 °C and proteinase k (400 μg/ml) for another 3 h at 50 °C. The DNA was extracted with 200 μl of phenol:chloroform:isoamyl alcohol (25:24:1) solution for 5 min and centrifuged at 14000 × *g* for 2 min. DNA was precipitated from aqueous phase by using 0.3 M sodium acetate at 4 °C overnight. Next day, the precipitate was centrifuged at 14000 × *g* for 10 min. The DNA pellet was washed in 80% alcohol, dried and dissolved in 40 μl of TE buffer. DNA was electrophoresed in 1.8% agarose gel at 70 V, stained with ethidium bromide and visualized in Bio-Rad gel documentation system.

### Apo BRDU assay

Apo BRDU assay was performed by using APO-DIRECT™ Kit (BD Pharmingen, Cat No- 51-6536AK) according to manufacturer’s protocol. Apo BRDU assay is used to detect single or double stranded breaks at DNA. In this assay, terminal deoxynucleotidyl transferase (TdT) enzyme catalyzes a template-independent addition of bromolated deoxyuridine triphosphates (Br-dUTP) to the 3′-hydroxyl (OH) termini of single or double stranded DNA. After incorporation, cells are incubated with FITC-labelled anti-BrdU monoclonal antibody, which bind to attached Br-dUTPs in the DNA and give fluorescence. Higher the Br-dUTPs fluorescence means more single or double stranded nicks in DNA. PANC-1 cells were treated with BA145 at 10, 20, 30 and 40 μM concentrations for 24 h. Cells were washed with PBS twice and fixed with 70% ice cold ethanol overnight. Next day, cells were centrifuged at 400 × g to remove ethanol and resuspended into wash buffer (Cat No-51-6548AZ) followed by TdT enzyme and FITC dUTP incubation as described in manufacturer’s protocol.

### Cell cycle analysis

Cells were treated with different doses of BA145 at different time intervals. Akt inhibitors were added 1 h before treatment. Cells were collected, washed with PBS twice and fixed overnight in 70% alcohol. Next day, cells are subjected to RNase digestion (200 μg/ml) at 37 °C for 1.30 h followed by incubation with PI (10 μg/ml) for another 30 min. Cells were acquired on FACS Calibur (BD, San Jose, CA) at FL2-H channel and cell cycle phase distribution were analyzed by using ModFit (Verity software house).

### Detection and Quantification of Acidic Vesicles

Acridine orange staining is used to stain acidic vesicles, a characteristic feature of autophagy. Cells were treated with BA145 (40 μM) at different time intervals. Inhibitors were added 1 h prior to BA145 treatment. Briefly, cells were incubated with acridine orange (1 μg/ml) for 10 min, washed with PBS twice and immediately examined under a fluorescent microscope. In case of monodansylcadaverine staining, cells were stained with monodansylcadaverine (50 μM) for 10 min, washed with PBS twice and observed under fluorescent microscope (Olympus IX70).

### Clonogenic assay

Cells were treated with BA145 in presence or absence of inhibitors at different concentrations and time intervals. Cells were trypsinized, viable cells were counted and 1000 cells were plated in 60 mm petridishes to determine the effect of treatment on clonogenic survival. Dishes were placed in the incubator for 18 days at 37 °C in 5% CO_2_ and 95% humidity. The colonies were fixed in 4% formaldehyde for 30 min and stained with 1% crystal violet to count colonies.

### Immunofluorescence and Confocal microscopy

PANC-1 cells were grown on coverslips and treated with different concentrations of BA145. Cells were fixed, stained and analyzed under the confocal microscope as earlier described^22^.

### Preparation of cell lysates and western blot analysis

Cells were lysed with cell lysis buffer (50 mM Tris HCl, 150 mM NaCl, 1% triton X-100, 5 mM EDTA, 30 mM Na_2_HPO_4,_ 0.1% SDS, 50 mM NaF, 0.5 mM NaVO_4,_ 2 mM PMSF and 1% protease inhibitor cocktail) for 40 min at 4 °C. After incubation, cells were centrifuged at 12000 × rpm for 15 min at 4 °C and supernatant were collected. Protein concentration was determined by Bradford assay and samples were mixed with equal amount of 2X sample buffer (100 mM Tris HCl, 4% SDS, 0.1% bromophenol blue, 20% glycerol and 200 mM β-mercaptoethanol). After mixing, samples were heated at 100 °C for 5 min and subsequently cooled on ice prior to loading onto respective polyacrylamide gels (6–15%). Proteins (40–70 μg) were separated by polyacrylamide gel electrophoresis at 75 V for 3 h. Proteins were then transferred to a 0.45 μm PVDF membrane (Millipore, USA) at 100 V for 90 min. The membrane was washed with wash buffer (1.21% Tris base, 8.7% NaCl, 1% Tween 20) for 10 min and blocked in blocking buffer (5% milk powder or 3% BSA in wash buffer) at room temperature. After 1 h, membrane was incubated with primary antibody over night at 4 °C. Next day, membrane was washed with wash buffer and incubated with the HRP-conjugated secondary antibody (anti-mouse or anti-rabbit diluted in blocking buffer). After 1 h membrane was given 3 washings with wash buffer of 15 min each. The protein were detected by using Immobilon Western Chemiluminescent HRP Substrate according to manufacturer protocols (Millipore, USA) and visualised by using Chemidoc XRS detection system (BioRad laboratories, USA) or by using CL-Xposure film. Protein expression were quantified by using myImageAnalysis^TM^ software from Thermo Fisher Scientific.

### mTOR Kinase assay

Inhibition of mTOR kinase activity by BA145 was performed by using K-LISA mTOR activity kit from Calbiochem (Cat#CBA055). It is an ELISA-based assay that utilizes a p70S6K-GST fusion protein as a specific mTOR substrate. The assay was performed according to the manufacturer’s protocol. Briefly, recombinant p70S6K-GST fusion protein (100 μl) was incubated in the glutathione coated 96-well plate for 1 h at room temperature. Meanwhile, 49 μl of mTOR protein was mixed with 1 μl of tested concentrations of BA145 or DMSO (negative control) and incubated on ice for 20 min. After 1 h, 50 μL mixture of mTOR protein and BA145 or DMSO and 50 μL of 2X kinase assay buffer (20 mM HEPES, 20 mM MnCl_2_, 20 mM β-Glycerophosphate, 2 mM EDTA, 100 mM ATP and 1 mM DTT) was added in the plate wells to initiate the kinase reaction. The plate was incubated for 30 min at room temperature. The kinase reaction was stopped by adding 10 μl kinase stop solution to each well. Next, 100 μl of anti-p70S6K-T389 (99 μL antibody diluent: 0.1 μL anti-p70S6kinase, threonine 389 antibody) was added for 1 h followed by incubation with 100 μl of HRP-conjugated antibody for another 1 h to detect the threonine 389- phosphorylated p70S6K. Absorbance was measured at 450 nm and 595 nm spectrophotometrically and the IC50 values were calculated by analysing non linear regression with variable slope using GraphPad Prism-5 software.

### Molecular Docking studies

All the molecular docking studies of BA145 with mTOR were carried out using the Schrodinger suite 2015 molecular modelling software. To perform molecular docking studies three reported co-crystallized X-ray structures of mTOR^49^ viz. 4JSX, 4JT6 and 4JT5 were downloaded from the Protein Data Bank (PDB). All these protein structures were prepared using default parameters of the protein preparation wizard of Schrodinger Suite. In this process, bond order was assigned, disulphide bonds were created and only those water residues were kept which were interacting with protein as well as with the heteroatom. The entire system was then optimized using OPLS2005 force field. Grids were generated at the binding site, identified on the bases of already co-crystallized ligand to the receptor using receptor grid generation module. The co-crystallized ligands were extracted and docked on this grid in order to standardize the docking protocol using the Glide module^50^ of Schrodinger software. It was found that the co-crystallized ligand of PDB Id 4JT6 attained the best docked conformation compared with the co-crystallized conformation with RMSD value < 0.3 A° at extra precision (XP) scoring function of Glide module. Thus, all the molecular docking studies of BA145 with mTOR were performed with XP scoring function. In order to provide some flexibility to the binding site residues, induced fit docking was performed.

### Statistical analysis

Data are presented as means of three similar experiments and the error bars represent the standard deviation (SD) between the experiments. Statistical analysis was done by using Bonferroni method and p value < 0.05 was considered to be significant with ***p < 0.001, **p < 0.01, *p < 0.05.

## Additional Information

**How to cite this article**: Pathania, A. S. *et al*. Interplay between cell cycle and autophagy induced by boswellic acid analog. *Sci. Rep.* **6**, 33146; doi: 10.1038/srep33146 (2016).

## Supplementary Material

Supplementary Information
