# Supplementary material for: Interplay between cell cycle and autophagy induced by boswellic acid analog
Source: Sci Rep. 2016 Sep 29;6:33146. doi: 10.1038/srep33146 (PMC5041107; doi:10.1038/srep33146)
Supplement: Supplementary Information [file srep33146-s1.pdf]

Supplementary Information

Interplay between cell cycle and autophagy induced by boswellic acid analog

Anup S Pathania ,Santosh K Guru, Suresh Kumar, Ashok Kumar, Masroor Ahmad, Shashi Bhushan, Parduman R Sharma, Priya Mahajan, Bhahwal A Shah, Simmi Sharma, Amit Nargotra, Ram Vishwakarma, Hasan Korkaya and Fayaz Malik

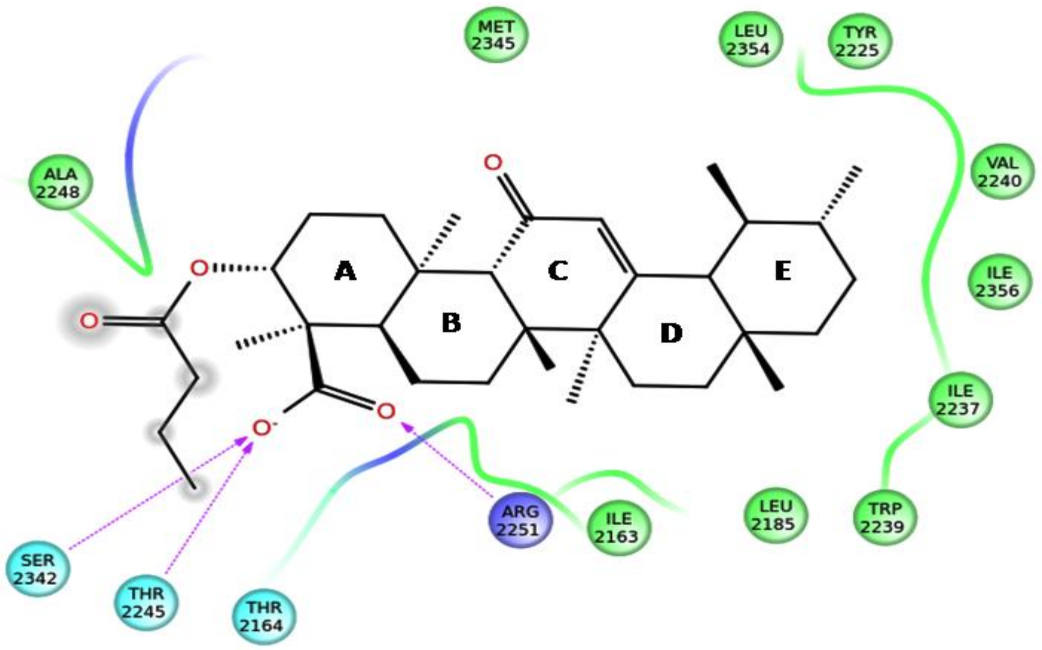

Figure. S1 2D Interaction diagram of BA145 with Mtor: here green, cyan and blue sphere indicates hydrophobic, polar and basic amino acids residues respectively of the protein within 3A^{o} distance from the centroid of the ligand.
